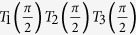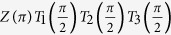# Photonic ququart logic assisted by the cavity-QED system

**DOI:** 10.1038/srep13255

**Published:** 2015-08-14

**Authors:** Ming-Xing Luo, Yun Deng, Hui-Ran Li, Song-Ya Ma

**Affiliations:** 1 Information Security and National Computing Grid Laboratory, Southwest Jiaotong University, Chengdu 610031, China; 2 School of Computer Science, Sichuan University of Science & Engineering, Zigong 64300, China; 3 School of Mathematics and Statistics, Henan University, Kaifeng 475004, China

## Abstract

Universal quantum logic gates are important elements for a quantum computer. In contrast to previous constructions of qubit systems, we investigate the possibility of ququart systems (four-dimensional states) dependent on two DOFs of photon systems. We propose some useful one-parameter four-dimensional quantum transformations for the construction of universal ququart logic gates. The interface between the spin of a photon and an electron spin confined in a quantum dot embedded in a microcavity is applied to build universal ququart logic gates on the photon system with two freedoms. Our elementary controlled-ququart gates cost no more than 8 CNOT gates in a qubit system, which is far less than the 104 CNOT gates required for a general four-qubit logic gate. The ququart logic is also used to generate useful hyperentanglements and hyperentanglement-assisted quantum error-correcting code, which may be available in modern physical technology.

Quantum algorithms have been explored to solve several difficult problems in terms of classical computers, e.g., large integer factoring^1^ and the quantum searching algorithm^2^. A full-scale quantum algorithm always requires joint control over multiple quantum systems, which currently represent challenging problems in experimental quantum physics. If the quantum circuit model^3^ is considered, any joint system evolutions may be synthesized with small-system evolutions, i.e., universal quantum gates^4^,^5^. Progress has been achieved for a variety of on universal quantum gates based on different physical architectures including the ions^6^,^7^, nuclear magnetic spins^8^,^9^, atoms^10^,^11^, and polarized photons^12^,^13^,^14^,^15^.

Large-dimensional states are necessary for quantum computation and for certain quantum information protocols^16^. Experimentally, the physical carrier of the qudit can be any *d*-dimensional quantum system. The high-dimensional quantum system is flexible in the storing and processing of quantum information^17^, such as the improvement of the channel capacity^18^,^19^, simplification of quantum gates^20^,^21^, and improvement of the communication security^22^,^23^,^24^. Moreover, the high-dimensional quantum system provides an alternate way for scaling quantum computation. Quantum algorithms with qubits typically require enforcing a two-level structure on atoms, ions or photon systems that naturally have many accessible degrees of freedom. Meaningful applications of qudits in quantum information always involves joint multiple qudits operations in a scalable manner.

In this paper, we consider the extension of universal qubit logic to the multivalue domain with hybrid quantum information systems, where the unit of memory is the ququart, a four-dimensional quantum system^16^,^17^. In photonic quantum information research, to encode a qudit, it is necessary to choose a multi-dimensional degrees of freedom (DOFs) of a single-photon, such as transverse momentum-position, the angular momentum, or time of arrival. Our four-dimensional quantum states are reformed from the natural two DOFs of photon in contrast to the symmetric primitive state of multiple photons under permutation invariance^25^,^26^. Although it is difficult to generate photonic nonlinear interactions with linear optics, however, recent hybrid systems (photon-matter)^27^,^28^,^29^,^30^ have been explored to effectively enable strong nonlinear interactions between single photons^31^ in the weak-coupling regime. The interface between special hybrid systems behaves in a manner similar to a beamsplitter using spin selective dipole coupling. Their optical selection rules are realized with a single-electron charged self-assembled GaAs/InAs quantum dot in a micropillar resonator^32^,^33^, which may be applied to construct universal qubit gates on photon systems with one degree of freedom^31^,^34^,^35^ or two freedoms^36^,^37^,^38^. We first present universal ququart gates with only one parameter^16^. And then, the hybrid systems are used to realize these universal ququart gates on the photonic ququart system with polarization and spatial mode freedoms. All the proposed schemes are applicable in larger-scale quantum algorithms because of the high fidelities and efficiencies of the present quantum techniques.

## Results

Our consideration of a qudit system is the four-dimensional quantum system (ququart system). Similar to the qubit system^3^, it is very difficult to realize the evolutions of the joint ququart systems by controlling multiple systems. Therefore, elementary logic gates^16^,^17^ are very useful for synthesizing any quantum transformation in *SU*(4^*n*^) derived from the *n*-ququart system evolution. We introduce some one-parameter universal ququart gates that are different from the multiple-parameters based quantum logic gates^16^ and are very simple to demonstrate in an experiment. These elementary gates may be implemented on a photon system with two DOFs, i.e., 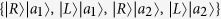 as the basis in four-dimensional space. Here, 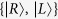 denotes the circular polarized basis, while 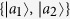 denotes the spatial modes. The primitive element is the quantum interface between a single photon and the spin state of an electron trapped in a quantum dot. These photonic ququart gates may be used for distributed quantum information processing.

### Cavity-QED system

**Figure 1:**
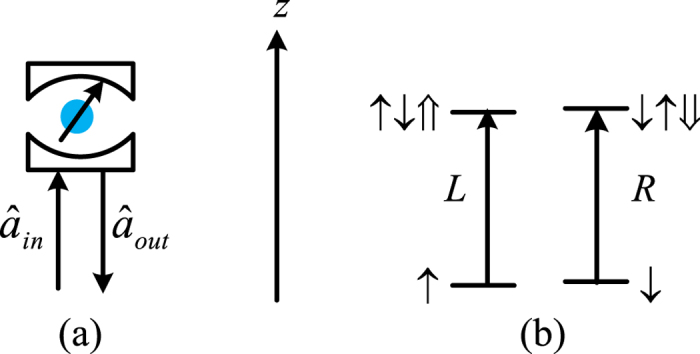
Schematic dipole spin-dependent transitions with circularly polarized photons. (**a**) A charged quantum dot inside a one-side micropillar microcavity interacting with circularly polarized photons. 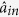 and 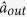 are the input and output field operators of the waveguide, respectively. (**b**) The optical selection rules due to the Pauli exclusion principle. *L* and *R* denote the left and right circular polarization respectively. ↑ and ↓ represent the spins of the excess electron. ↑↓⇑ and ↓↑⇓ denote the negatively charged excitons.

The cavity-QED system used in our proposal is constructed by a singly charged In(Ga)As quantum dot located in the center of a one-sided optical resonant cavity^27^,^28^,^29^,^30^,^31^, as shown in Fig. 1. The single-electron states have *J*_*z*_ = ±1/2 spin (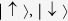) and the holes have *J*_*z*_ = ±3/2 (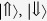). Two electrons form a singlet state and therefore have a total spin of zero, which prevents electron spin interactions with the hole spin. The photon polarization is commonly defined with respect to the direction of propagation, whereas the absolute rotation direction of its electro-magnetic fields does not change. The input-output relation of this one-sided cavity system can be calculated from the Heisenberg equation^36^,^37^,^38^,^39^,^40^,^41^,^42^ of motion for the cavity field operator and dipole operator as follows:


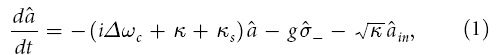



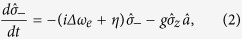



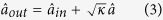


where Δ*ω*_*c*_ = *ω*_*c*_ − *ω*, Δ*ω*_*e*_ = *ω*_*e*_ − *ω*. *ω*_*c*_, *ω* and *ω*_*e*_ are the frequencies of the cavity mode, the input probe light, and the dipole transition, respectively. *g* is the coupling strength between the cavity and dipole. *η*, *κ*, and *κ*_*s*_ are the decay rates of the dipole, the cavity field, and the cavity side leakage mode, respectively. If the dipole stays in the ground state most of the time^39^,^40^,^41^,^42^, then by adapting the frequencies of the light and the cavity mode, the interaction of a single photon with a cavity-QED system can be described as the following transformation


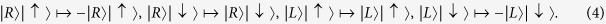


### Universal ququart logic gates

Consider the following gates


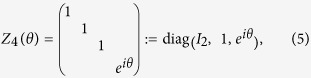



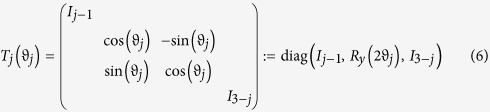


with *j* = 1, 2, 3, which are operated on the four-dimensional Hilbert space (ququart system). diag(·,·) denotes the diagonal matrix. *R*_*y*_(2*ϑ*_*j*_) denote real rotation matrices with phases *ϑ*_*j*_, and *I*_*k*_ represents the identity operation in *SU*(*k*) for each *k* ≥ 1. {*Z*_4_(*θ*), *T*_*j*_(*ϑ*_*j*_), *j* = 1, 2, 3} as a set of one-parameter transformations may be sufficient to simulate all single-ququart unitary transforms. The proof of the idea is derived in Ref. 16. In fact, for a logic four-dimensional basis 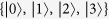, note that


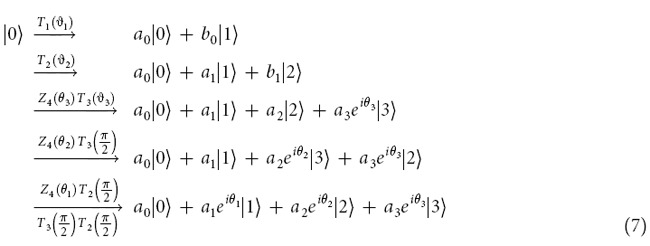


where 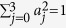, 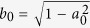, 
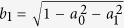
, 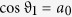, 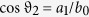, 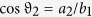. In other words, the ququart system 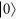 may be changed into an arbitrary ququart system 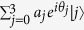. For simulating the evolution of a joint system, similar to the qubit case^3^,^4^, elementary logic gates should be constructed. In detail, we define controlled ququart gates as follows:


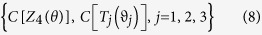


acting on a two-ququart system, where *C*[*Z*_4_(*θ*)] and *C*[*T*_*j*_(*ϑ*_*j*_)] are defined as


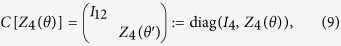



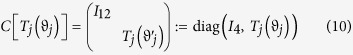


which indicates that the ququart operation *Z*_4_(*θ*) or *T*_*j*_(*ϑ*_*j*_) is performed on the target ququart system if the controlling ququart system is in the state 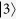. Generally, the set


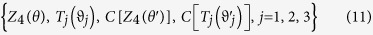


is a set of simplified universal ququart gates for synthesizing the joint system operations in *SU*(4^*n*^). In fact, from the representation theory of the unitary matrix and eigenoperator decompositions^16^, all *n*-ququart unitary operations **U** ∈ *SU*(4^*n*^), and there exist 4^*n*^ different eigenstates 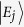 of **U**, 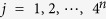, with corresponding eigenvalues 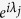. Here, each eigenstate is represented as


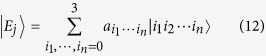


From the unitary matrix representation theory **U** is rewritten as


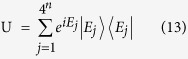


with eigenoperators


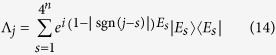


thus generating a phase *λ*_*j*_ of 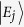 without affecting any other eigenstates^16^. The followed proof synthesizes these eigenoperators from


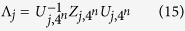


Here, 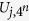 and 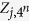 are the 4^*n*^-dimensional analogs of the ququard operation in Equation (7) and *Z*_4_(*θ*). 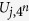 only transforms the *j*-th eigenstate to 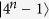, i.e.,


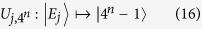


*Z*_*j*,*N*_ changes the phase of 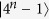 with the *j*-th eigenphase, leaving all other computational states unchanged,


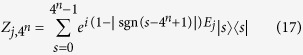


where sgn is the sign function. With involved computations similar to these in Ref. 16, one can prove that 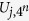 and 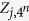 may be realized with logic gates in equation (11). Thus all *n*-ququart unitary operations **U** ∈ *SU*(4^*n*^) may be synthesized with ququart operations {*Z*_4_(*θ*), *T*_*j*_(*ϑ*_*j*_)} and controlled ququart operations {*C*[*Z*_4_(*θ*′)], *C*[*T*_*j*_(*ϑ*_*j*′_)]}. However, different from the multiple-parameter ququart gates^16^, all the universal ququart gates are of one-parameter and easy to be realized in an experiment.

### Photonic universal ququart logic gates

The ququart basis is defined as 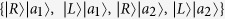. Note that all ququart logic gates *Z*_4_(*θ*) and *T*_*j*_(*ϑ*_*j*_) are also two-qubit logic gates. The ququart rotation *Z*_4_(*θ*) is a controlled phase rotation gate on a two-qubit system. The ququart gates *T*_1_(*ϑ*_1_) and *T*_3_(*ϑ*_1_) are controlled rotations on a two-qubit system. The second qubit is the controlling qubit for *T*_1_(*ϑ*_1_) while the first qubit is the controlling qubit for *T*_2_(*ϑ*_1_). *T*_2_(*ϑ*_1_) is a general swapping gate on a two-qubit system. Thus, they are easily synthesized with the universal qubit gates, such as the controlled not gate (CNOT) and single qubit rotations^3^,^4^. These universal qubit gates may be realized on the photon with the polarization and spatial mode DOFs^35^,^36^,^37^,^38^.

**Figure 2:**
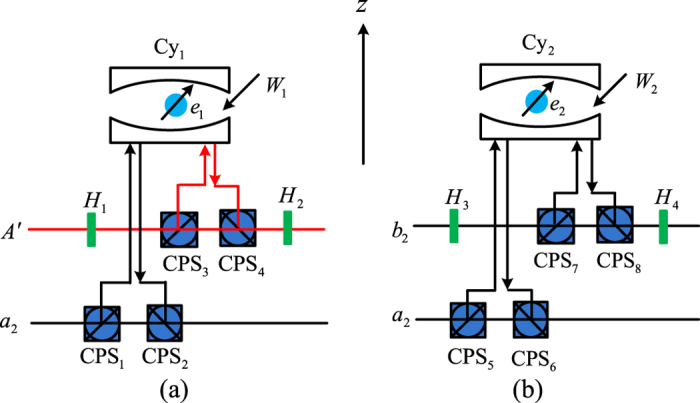
Schematic circuit of elementary ququart gates. (**a**) Schematic hybrid controlled not (CNOT) gate on a ququart-qubit photon system. (**b**) Schematic controlled phase *C*[*Z*_4_(*π*)] on a two-ququart photon system. CPS_*j*_ represent polarizing beamsplitters in the circular basis, which transmit 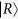 and reflect 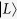. *W*_*j*_ represent the Hadamard operations 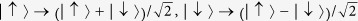 on the excess electron spins *e*_*j*_. *H*_*j*_ represent half-wave plates (HWP) to perform the Hadamard operation 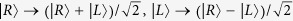 on the polarization DOF of a photon.

Figure 2 shows how the interface between the input photon and an electron spin confined in a quantum dot embedded in a microcavity can be used to construct two-ququart gates defined in equation (8). The auxiliary electron spins are in the states 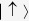. Two input ququart photons *A* and *B* are in the states


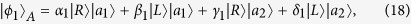



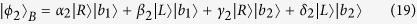


respectively.

The controlled ququart gate *C*[*Z*_4_(*θ*)] is realized as follows. The first step is to complete a hybrid CNOT gate on the polarization DOF of the photon *A* and the auxiliary photon *A*′ (red line) in the state 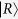, shown in Fig. 2(a). After a Hadamard operation *W*_1_ on the electron spin *e*_1_, the photon *A* from the spatial mode *a*_2_ passes through *CPS*_1_, *Cy*_1_, *CPS*_2_, sequentially. With a Hadamard operation *W*_2_ on the electron spin *e*_1_, the joint system of the photon *A* and the spin *e*_1_ is changed from 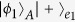 into


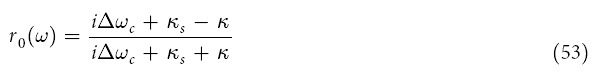


from a hybrid CNOT gate on the polarization freedom of the ququart photon and the spin *e*_1_,


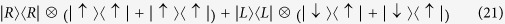


The followed circuit consisting of the *H*_1_, *CPS*_3_, *Cy*_1_, *CPS*_4_, and *H*_2_ represents a hybrid CNOT gate on the electron spin *e* and the auxiliary photon *A*′ as follows


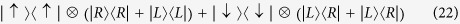


which may change the joint system in the state 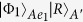 into


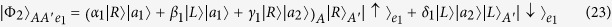


The quantum spin *e*_1_ in the entanglement 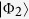 shown in equation (23) may be measured under the basis 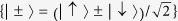 in order to achieve a ququart-qubit photon system


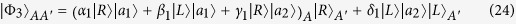


Here, a Pauli phase flip (*σ*_*Z*_) is performed on the polarization DOF of the photon *A* from the spatial mode *a*_2_ for the measurement outcome 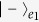. Thus, Fig. 2(a) has realized a hybrid CNOT gate on the ququart-qubit photon system with the matrix representation diag(*I*_6_, *σ*_*X*_). Similarly, for the photon *B* and an auxiliary photon *B*′ in the state 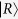, by using the circuit shown in Fig. 2(a), the joint system of the photon *B* and auxiliary photon *B*′ is changed from the state 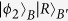 into


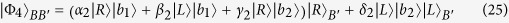


Note that 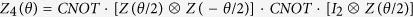, which may be redefined as the controlled rotation gate on the two-qubit photonic system *A*′ and *B*′ with two CNOT gates^34^,^35^. The joint system of four photons *A*, *B*, *A*′ and *B*′ in the state 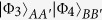 will collapse into


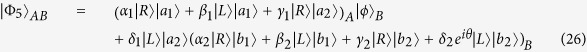


after the measurements of the photons *A*′ and *B*′ under the basis 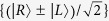. Here, the Pauli phase flip *σ*_*Z*_ is performed on the polarization DOF of the photon *A*(*B*) from the spatial mode *a*_2_(*b*_2_) for the measurement outcome 
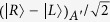
 or 
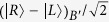
. *Z*(*θ*/2) = diag(1, *e*^*iθ*/2^) is a general qubit phase gate. Therefore, the controlled ququart rotation *C*[*Z*_4_(*θ*)] = diag(*I*_14_, 1, *e*^*iθ*^) is realized with eight CNOT gates on a hybrid two-qubit system (spin and photon or photon and spin), as shown in Table 1.

To realize the controlled ququart rotation *C*[*X*_*j*_(*ϑ*_*j*_)], consider the special controlled-ququart flip gate *C*[*Z*_4_(*π*)] without two auxiliary photons, shown in Fig. 2(b). One hybrid CNOT gate is performed on the photon *A* and an auxiliary spin *e*_2_ in the state 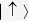 with *W*_3_, *CPS*_5_, *Cy*_2_, *CPS*_6_, and *W*_4_. The other hybrid CNOT performed on the electron spin *e*_2_ and the photon *B* is realized with *H*_3_, *CPS*_7_, *Cy*_2_, *CPS*_8_, and *H*_4_. The joint system of two photons *A* and *B* may be changed from the initial state 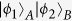 into


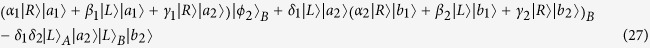


after measuring the spin *e*_2_ under the basis 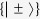, where a Pauli phase flip *σ*_*Z*_ is performed on the photon *A* from the spatial mode *a*_2_ for the measurement outcome 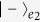. Thus, a controlled-ququart flip gate *C*[*Z*_4_(*π*)] has been realized on the photons *A* and *B*.

With the circuit the two-ququart gate *C*[*Z*_4_(*π*)], the controlled ququart gates *C*[*T*_*j*_(*ϑ*_*j*_)] may be realized with the following decomposition


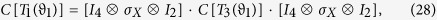



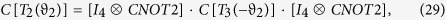



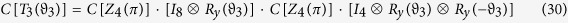


Here, CNOT2 denotes the CNOT gate with the second input qubit being the controlling qubit. The costs of hybrid CNOT gates are shown in Table 1. They are far less than 104 CNOT gates required for general unitary operations acting on four-qubit system^5^.

### Hyperentanglement preparation

**Figure 3:**
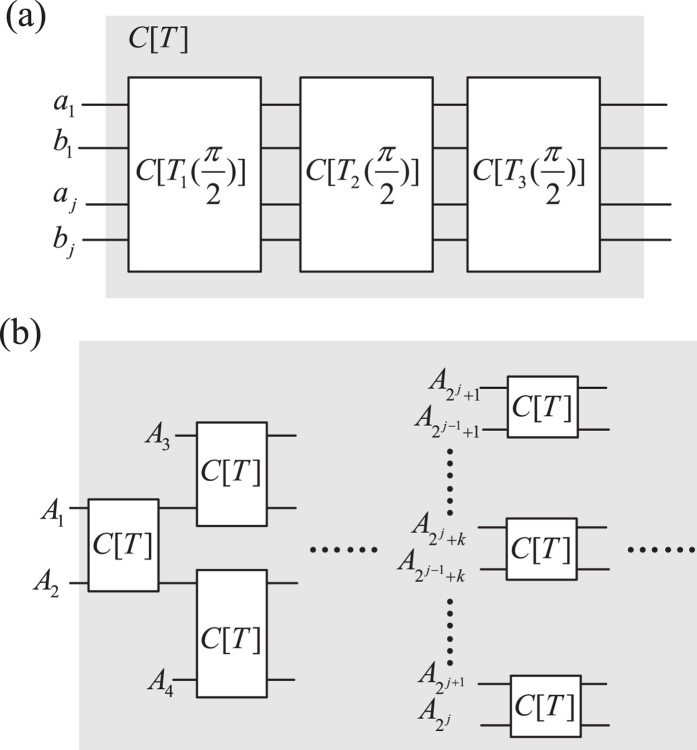
Schematic ququart cat-state preparation. (**a**) Elementary ququart copying operation on two-ququart system, (**b**) Parallel implementation of *n*-ququart cat state with 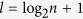 time slices.

Hyper-entangled photonic states^43^ have been experimentally realized and shown to offer significant advantages in quantum information processing^19^,^22^,^23^,^24^,^25^,^44^. Our first scheme is for the cat state in the form


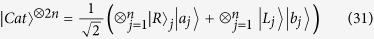


where *R* and *L* denote right- and left-circular polarization and *a*_*i*_, *b*_*j*_ label two orthogonal spatial modes of the photons. This state exhibits maximal entanglement between all photon polarizations and spatial qubits, and has been experimentally realized with *n* = 10^45^ from the spontaneous parametric down-conversion and pseudo-single photon source. Here, we present a general *n*-ququart cat state with the present elementary ququart gates in Equation (11), shown in Fig. 3. Note that from Fig. 3(a)


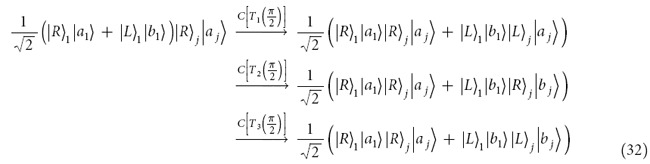


which has realized the ququart copying operation on the photon *A*_1_ in the state 
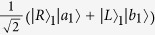
 and the photon *A*_*j*_ in the state 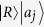. With this elementary circuit, by using the parallel implementation in Fig. 3(b), 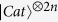 can be easily generated.

**Figure 4:**
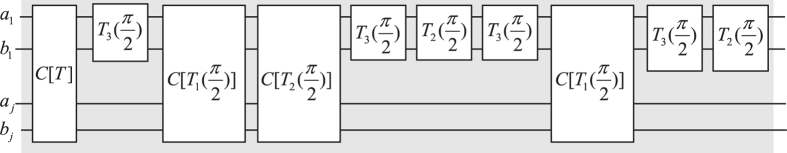
Schematic ququart cluster-state preparation. *C*[*T*] is defined in Fig. 3(a).

The second hyper-entangled photonic state is the *n*-ququart cluster state,


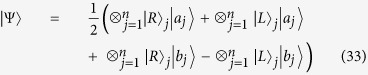


which may be used for one-way quantum computing^44^ when *n* = 2. Our generation circuit is shown in Fig. 4. It easily follows that


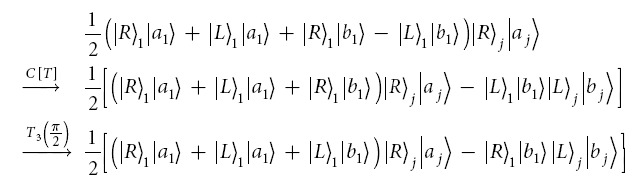



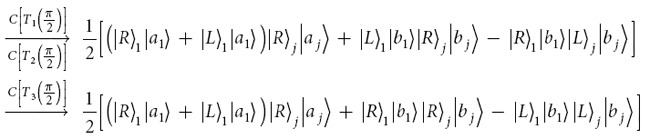



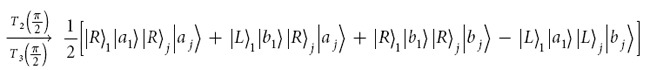



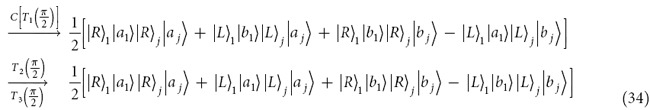


which has realized the ququart copying operation on the photon *A*_1_ in the state 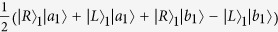 and the photon *A*_*j*_ in the state 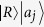. With this elementary circuit, similar to Fig. 3(b), 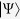 can be easily generated. Moreover, if the first photon is in the initial state 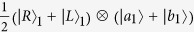, from Fig. 4, it can follow another hyperentangled *n*-photon GHZ state, which can be written as


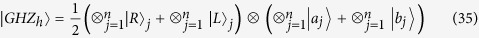


### Hyperentanglement-assisted quantum error-correcting code

The code is hyperentanglement assisted because the shared entanglement resource is a photonic state hyperentangled in the polarization and spatial mode. It is possible to encode, decode, and diagnose channel errors using cavity-QED techniques. This code may be used to correct the polarization flip errors and is thus suitable only for a proof-of-principle experiment. The quantum channel is constructed with the following hyperentanglement


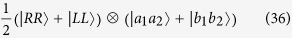


If we only change the polarization DOF of the first photon in this state according to the four Pauli operators, it then follows four hyperentangled states:


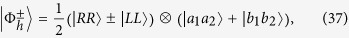



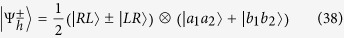


**Figure 5 f5:**
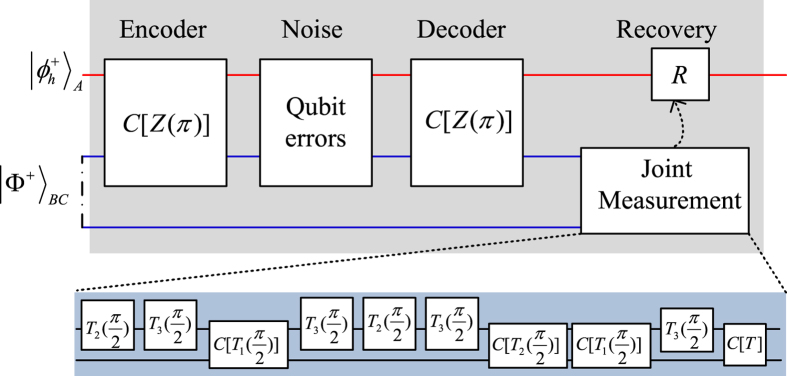
Schematic hyperentanglement-assisted quantum code. Blue lines are a hyperentangled state 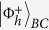. The input photon *A* is in the state 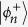. The encoding circuit consists of one controlled-phase gate *C*[*Z*(*π*)]. The polarization-error may be derived in a noisy environment or noisy quantum channel for quantum super-dense coding. The joint measurement is completed with a hyper-Bell state analysis to determine the error syndrome. The recovery operations *R* is dependent of the measurement outcomes shown in Table 1.

These states may be rewritten in terms of the single-photon polarization-spatial mode states


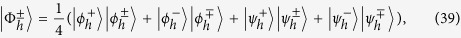



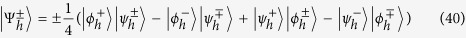


where


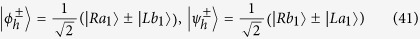


with single photon basis states 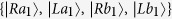. Figure 5 shows our hyperentanglement-assisted quantum code. As an example, the input state can be 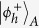. The encoding circuit consists of one controlled-sign gate *C*[*Z*(*π*)] such that the joint state is the following normalized encoded state


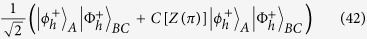
[Fig f5]

If the noisy environment has not introduced polarization errors on the photons *A* and *B*, after the decoding circuit (same as the encoding circuit), and the resulting decoded state is defined by


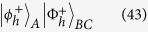


For polarization errors, the relationship between the syndrome and errors is shown in Table 2. Here, we encode one of four classical messages (two classical bits) by applying one of four transformations to the first photon of 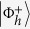: (1) the identity, (2) Pauli phase flip 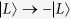 on the polarization DOF which corresponds to 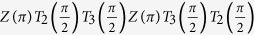 realizing 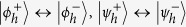, (3) Pauli flip 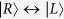 on the polarization DOF, which corresponds to 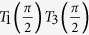 realizing 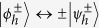, or (4) both Pauli phase flip and Pauli flip. The result is to transform the original state 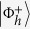 to one of the four states 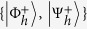.

## Discussion

In the experiment, the ququart gates’ fidelities are defined by 
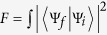
, where 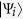 and 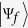 are the final states under the ideal condition and the real situation with side leakages, respectively. In the resonant condition, if the cavity side leakage is considered, then the optical selection rules in equation (4) from the cavity-QED system is given by:


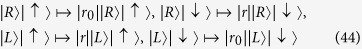


where only real reflection coefficients |*r*_0_| and |*r*| are considered. To estimate the photon scattering probability, the area of the light beam, 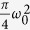 is compared to the absorption cross section of the spin^39^,^46^, 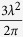 with the optical wavelength *λ*. Deterministic spin-photon interaction requires 
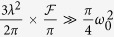
 with the number of bounces 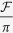
35,47. The resonator quality 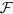 is characterized by its finesse 
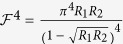
, which depends on the reflectivity of the mirrors, *R*_1_ and *R*_2_. The spin-cavity coupling constant *g* is determined by the electric dipole matrix element *μ* of the transition from the (coupled) ground state to the excited state and by the electric field *E* of a single photon in the mode volume *V* of the resonator^39^,^46^,^47^,^48^,^49^,^50^:


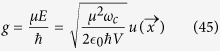


*∈*_0_ is the permittivity of free space, and 
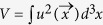
 is the integral over the dimensionless electric-field mode function 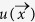 of the resonator, normalized to one at the field maximum. The decay rate *η* of the dipole is formed as


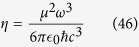


The decay rate *κ* of the cavity field is defined as^39^


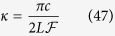


where *c* is the speed of light and *L* is the resonator length. The deterministic spin-photon interaction condition leads to the strong coupling


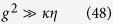


In this strong-coupling regime (coupling constant *g* = 2*π* ⋅ 6.7 MHz, atomic dipole decay rate *ς* = 2*π* ⋅ 3 MHz, cavity field decay rate *κ* = 2*π* ⋅ 2.5 MHz), with a coupled atom, the phase shift is realized to be zero^35^. The resulting conditional phase shift is the basis for the realization of robust quantum gates^31^,^48^. This gate, as a primitive gate for photonic qubit-based computation, is also an elementary gate for our universal ququart gates presented in Table 1. In our setup, the input photon is either transmitted through the cavity mirror with rate *κ* or lost with rate *κ*_*s*_. *κ*_*s*_ gives^35^


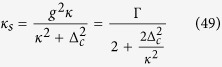


with the relaxation time Γ = 2*g*^2^/*κ* of the dipole. The decay into the resonator mode is suppressed by increasing the detuning Δ_*c*_ between spin and cavity. On resonance, the radiative interaction of the spin with the environment is then dominated by the cavity mode rather than the free-space modes. A recent experiment shows that an almost tenfold reduction of the spin excited state lifetime is observed^50^.

**Figure 6 f6:**
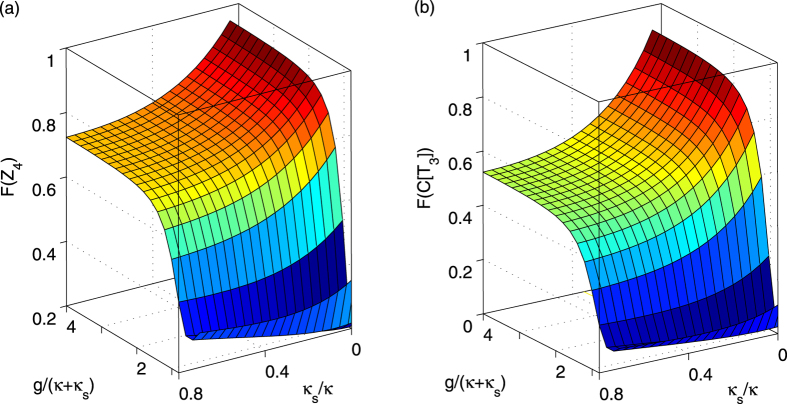
Average fidelities of ququart gates. (**a**) The average fidelity *F*(*Z*_4_) of the ququart gate *Z*_4_(*θ*) versus the normalized coupling strengths *κ*_*s*_/*κ* and *g*/(*κ* + *κ*_*s*_). (**b**) The average fidelity *F*(*C*[*T*_3_]) of the controlled ququart gate *C*[*T*_3_(*ϑ*)] versus the normalized coupling strengths *κ*_*s*_/*κ* and *g*/(*κ* + *κ*_*s*_). The coupling strength is defined by *η* = 0.2*κ*_*s*_. The average fidelity is computed as the average of random *θ* and *ϑ*.

**Figure 7 f7:**
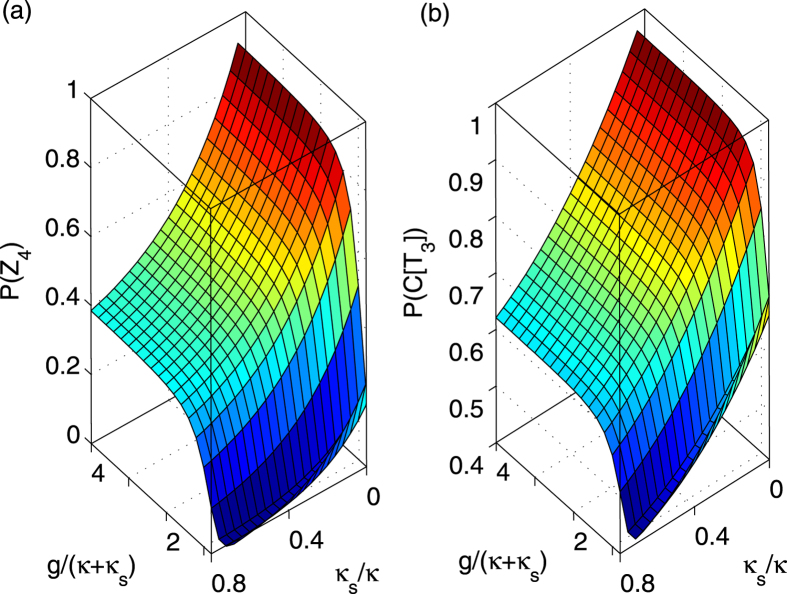
Average efficiencies of ququart gates. (**a**) The efficiency *P*(*Z*_4_) of the ququart gate *Z*_4_(*θ*) versus *κ*_*s*_/*κ* and *g*/(*κ* + *κ*_*s*_). (**b**) The efficiency *P*(*C*[*T*_3_]) of the controlled ququart gate *C*[*T*_3_(*ϑ*)] versus *κ*_*s*_/*κ* and *g*/(*κ* + *κ*_*s*_). Here, *η* = 0.2*κ*_*s*_.

Based on the new rule in equation (44), the fidelities and efficiencies of our ququart gates *Z*_4_(*θ*) and *C*[*T*_3_(*ϑ*)] are calculated, as shown in Figs 6 and 7, respectively. The other ququart gates may be easily calculated using equation (28) and equation (29). The efficiency is defined as the probability of the two photons to be detected after the logic operation. To demonstrate our fidelities and efficiencies, these evaluations are based on the relative coupling strength and relative decay ratios. When 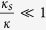, i.e,


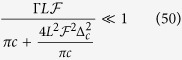
[Fig f6][Fig f7]

The above may be realized by enhancing the resonator quality 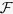, increasing the resonator length *L* or detuning Δ_*c*_. In this case, high fidelities and efficiencies may be achieved, even in the weakly coupling regime 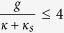. If *κ*_*s*_ ≪ *κ* is not satisfied, then high fidelities and efficiencies require strong coupling *g*^2^ ≫ *η*(*κ* + *κ*_*s*_) from equation (48). A recent experiment^39^ has raised the coupling from 0.5 (the quality factor *Q* = 8800^41^) to 2.4 (the quality factor *Q* = 40000^40^) by improving the sample designs, growth, and fabrication in 1.5 *μm* micropillar microcavities. For our ququart gates, the fidelities are greater than 93.5% and the efficiencies are greater than 64.6% for 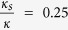 and 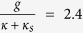. In the experiment, to derive a critical photon number, which determines the number of photons required to significantly change the radiation properties of the spin, the rate of spontaneous emission 2*γ* must be compared to the rate of stimulated emission per photon, 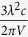.

In the poor cavity limit, the coupling between the radiation and the dipole can change the cavity reflection and transmission properties^49^,^50^, which allows for quantum applications in the weak coupling regime. In general the difference between the transmission for the uncoupled and coupled cavity can be increased by reducing the cavity losses and increasing the Purcell factor and the dipole lifetime. The preparation and the Hadamard operation of an electron spin may be realized using nanosecond electron spin resonance microwave pulses^47^. The ground state degeneracy, with Zeeman splitting less than the photon bandwidth, must be restored in the implementation of quantum information protocols^46^. Quantum optical applications, such as the photon entangling gate and quantum computation, require the dephasing time being typically within the range of 5–10 ns. The electron spin coherence time can be extended to *μ*s using spin echo techniques^51^,^52^,^53^,^54^,^55^,^56^ to protect the electron spin coherence with microwave pulses. The optical coherence time of an exciton is ten times longer than the cavity photon lifetime^57^,^58^, with which the optical dephasing only reduces the fidelity by a few percent. The hole spin dephasing is dominant in the spin dephasing of the dipole, and it can be safely neglected with the hole spin coherence time being three orders greater than the cavity photon lifetime^59^.

In conclusion, we introduced one-parameter universal ququart gates for *SU*(4^*n*^) based on the four-dimensional Hilbert space. These elementary gates are simpler than the multi-parameter ququart gates^16^. Moreover, in contrast to their iron-based realizations, our gates may be implemented on a photon system with two DOFs. The primitive element is the quantum interface between a single photon and the spin state of an electron trapped in a quantum dot, based on a cavity-QED system. Because of the superiority of the proposed gates regarding transmission, these photonic ququart gates may be used for distribution quantum information processing. Compared with previous qubit gates on the one DOF of a two-photon system^31^,^34^,^38^ or the hybrid gates on the photon and stationary electron spins^35^, our gates are created on two photons of two DOFs simultaneously. Different from previous CNOT gates on the same DOF of a two-photon system^36^, or CNOT gates on the different DOFs of a photon system^37^, our ququart gates require four qubits (a pair of two-DOFs). All elementary ququart gates cost no more than eight hybrid CNOT gates for a two-qubit system, which is far less than the 104 CNOT gates required for a general four-qubit gate. These elementary ququart gates are ultimately realized on the photon system for multi-system hyperentanglement, such as the cat state^45^, cluster state^43^, or GHZ state. The present photonic ququart logic may be applied to large-scale quantum computation.

## Method

The cavity-QED system used in our proposal may be constructed as a singly charged In(Ga)As quantum dot located in the center of a one-sided optical resonant cavity^29^,^30^,^31^,^32^ to achieve maximal light-matter coupling, as shown in Fig. 1. Microdisks and photonic crystal nanocavities may be used to produce single-photon sources and to study the Purcell effect in the weak-coupling regime and the vacuum Rabi splitting in the strong coupling regime^40^. If the quantum dot is singly charged, i.e., a single excess electron is injected, the optical excitation can create a negatively charged exciton (*X*^−^). The single-electron states have *J*_*z*_ = ±1/2 spin (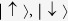) while the holes have *J*_*z*_ = ±3/2 (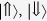). The two electrons form a singlet state and therefore have a total spin of zero, which prevents electron spin interactions with the hole spin. Photon polarization is commonly defined with respect to the direction of propagation, whereas the absolute rotation direction of its electromagnetic fields does not change. We will therefore label the optical states by their circular polarization (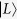 and 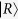 for left- and right-circular polarization respectively). Due to Pauli’s exclusion principle, *X*^−^ shows that spin-dependent optical transitions^39^,^40^ [see Fig. 1(b)], a negatively charged exciton 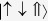 or 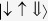 may be created by resonantly absorbing 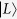 or 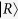
39,40. Due to this spin selection rule, the photon pulse encounters different phase shifts after reflection from the *X*^−^-cavity system when *X*^−^ strongly couples to the cavity.

In the frame rotating with the cavity frequency *ω*_*c*_, the input-output relation of this one-sided cavity system can be calculated from the Heisenberg equation^39^,^40^ of motions for the cavity field operator 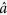 and dipole operator 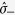 shown in equations (1), (2) and (3). In the approximation of weak excitation, i.e., 〈*σ*_*z*_〉 ≈ −1, when both the adiabatic condition (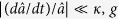) and the strong coupling condition (*g*^2^ ≫ *κγ*) are satisfied, the spin always stays in the steady state^39^,^40^,^41^,^42^,^43^,^44^. From 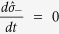 and 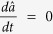, it follows that


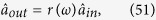


where the reflection coefficient


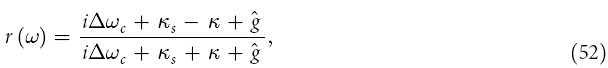



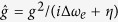
 with the frequency detuning of Δ*ω*_*e*_ between the photon and the dipole transition. *g* is the coupling strength between the cavity and dipole transition. *η*, *κ*, and *κ*_*s*_ are the decay rates of the dipole transition, the cavity field, and the cavity side leakage mode, respectively. In the following, we consider the case of a dipole tuned into resonance with the cavity mode (Δ*ω*_*e*_ = 0), probed with the resonant light (*g* = 0, *η* → 0). If the radiation is not coupled to the dipole transition (*g* = 0, *η* → ∞), then the reflection coefficient in equation (1) becomes


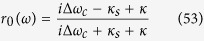


Thus, the optical process based on the spin-dependent transition is obtained^37^,^38^. The reflection coefficients can reach |*r*_0_(*ω*)| ≈ 1 and |*r*_*h*_(*ω*)| ≈ 1 when the cavity side leakage *κ*_*s*_ is negligible. If the linearly polarized probe beam in the state 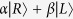 is placed into a one-sided cavity-QED system with the superposition spin in the state 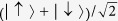, then the joint system consisting of the photon and the electron spin after reflection is


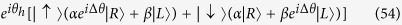


where Δ*θ* = *θ*_0_ − *θ*_*h*_ with *θ*_0_ = arg[*r*_0_(*ω*)] and *θ*_*h*_ = arg[*r*_*h*_(*ω*)]. By adjusting the frequencies of the light and the cavity mode, the phase difference Δ*θ* for the left- and right-circular polarized photons may reach up to *π*^33^. From equation (48), the interaction of a single photon with a cavity-QED system can be described as in equation (4)^60^,^61^.

## Additional Information

**How to cite this article**: Luo, M.-X. *et al*. Photonic ququart logic assisted by the cavity-QED system. *Sci. Rep*. **5**, 13255; doi: 10.1038/srep13255 (2015).

## Figures and Tables

**Table 1:** The cost of CNOT on a hybrid two-qubit system (spin and photon or photon and spin) for each elementary ququart logic gate.

Ququart gates	*Z*_4_(*θ*)	*T*_1_(*ϑ*_1_)	*T*_2_(*ϑ*_2_)	*T*_3_(*ϑ*_2_)
Hybrid CNOT cost	4	2	6	2
Controlled ququart gates	*C*[*Z*_4_(*θ*)]	*C*[*T*_1_(*ϑ*_1_)]	*C*[*T*_2_(*ϑ*_2_)]	*C*[*T*_3_(*ϑ*_3_)]
Hybrid CNOT cost	8	4	8	4

**Table 2:** The recovery operations dependent of the results of hyper-Bell state analysis.

Errors	*I*	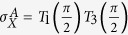	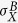	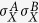
Hyper-Bell state	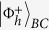	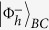	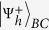	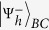
Measure state	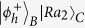	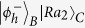	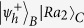	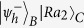
Recovery *R*	*I*	*Z*(*π*)